# Gut microbiota dysbiosis in autism spectrum disorder: 10 years of progress on compositional alterations, metabolic/immune mechanisms, and therapeutic strategies

**DOI:** 10.3389/fnins.2026.1873864

**Published:** 2026-07-28

**Authors:** Yan Zeng, Fengyang Wang, Silu Li, Qianfang Liu, Lin Liu, Bin Song

**Affiliations:** 1 Department of Pediatrics, Deyang Key Laboratory of Birth Defects Prevention and Control, Deyang People’s Hospital, Deyang, Sichuan, China; 2 Department of Nephrology, Deyang People’s Hospital, Deyang, Sichuan, China

**Keywords:** autism spectrum disorder, dysbiosis, fecal microbiota transplantation, gut microbiota, gut–brain axis, metabolomics, neuroinflammation, probiotics

## Abstract

Autism spectrum disorder (ASD) is a common neurodevelopmental condition frequently accompanied by gastrointestinal symptoms, pointing to a potential role of the gut microbiota–brain axis. To explore this connection, the present review synthesizes findings from studies published between 2016 and 2026, including observational studies, meta-analyses, animal experiments, and clinical trials, with the aim of characterizing gut microbiota alterations in ASD, elucidating underlying mechanisms, and evaluating emerging therapeutic strategies. Across diverse populations, the most consistent microbial signatures in ASD include reduced abundances of *Bifidobacterium* and *Akkermansia muciniphila*, together with increased abundances of *Clostridium*, *Bacteroides*, and *Escherichia–Shigella*; however, geographic, age-, and sex-specific variations exist. In addition to bacterial changes, the gut virome and mycobiome are also perturbed, as evidenced by enrichment of *Candida albicans* and *Clostridium phages*. Mechanistically, these alterations are linked to reduced short-chain fatty acids (especially *butyrate*), disrupted tryptophan–serotonin metabolism, and elevated neuroinflammatory cytokines (e.g., TNF-α, IL-6). Causal evidence from animal models using fecal microbiota transplantation further demonstrates that ASD microbiota can directly induce autistic-like behaviors. Building on this causal link, early-phase clinical trials indicate that fecal microbiota transplantation, probiotics, prebiotics, and dietary interventions (e.g., ketogenic diet) can improve both gastrointestinal and behavioral symptoms, although larger double-blind, placebo-controlled trials are needed to confirm efficacy. Furthermore, multi-omics integration and host epigenetic signatures show promise for developing non-invasive diagnostic biomarkers. In conclusion, gut dysbiosis plays a causal role in ASD pathophysiology, and microbiome-based interventions represent a rational and potentially transformative therapeutic avenue.

## 1 Introduction

Autism spectrum disorder (ASD) is a heterogeneous neurodevelopmental condition with an estimated prevalence of 0.6–1.7% in children worldwide (Iglesias-Vázquez et al., 2020). It is characterized by persistent deficits in social communication and interaction, as well as restricted, repetitive patterns of behavior, interests, or activities. The etiology of ASD remains poorly understood but involves a complex interplay of genetic and environmental factors (Beopoulos et al., 2021; Kong et al., 2019; Liu et al., 2021).

Beyond core symptoms, gastrointestinal (GI) disturbances are highly prevalent in children with ASD, affecting up to 70% of individuals (Kong X. et al., 2021). Common symptoms include chronic constipation, diarrhea, abdominal pain, and bloating (Ding et al., 2021; Iglesias-Vázquez et al., 2020). The severity of GI symptoms often correlates positively with the severity of autistic behaviors, suggesting a shared underlying mechanism (Ding et al., 2021). The concept of the microbiota–gut–brain axis provides a biological framework linking gut microbes to central nervous system function through neural, hormonal, and immune pathways (Beopoulos et al., 2021; Parsons et al., 2020).

Several lines of evidence support a role for gut dysbiosis in ASD: (i) children with ASD frequently exhibit altered gut microbiota composition compared to typically developing (TD) controls; (ii) germ-free mice colonized with ASD microbiota develop ASD-like behaviors; (iii) interventions that modulate gut microbes, such as fecal microbiota transplantation (FMT) or probiotics, can improve both GI and behavioral symptoms (Goo et al., 2020; Kang et al., 2017; Xiao et al., 2021). However, there is no universal microbial signature for ASD. Meta-analyses have reported inconsistent changes in specific taxa, partly due to differences in age, diet, geographic location, sequencing methods, and study design (Jurek et al., 2021; Kadiyska et al., 2025).

In this review, we systematically searched PubMed, Web of Science, Scopus, and China National Knowledge Infrastructure for studies published during 2016-2026. The following keywords were used: (“autism spectrum disorder” OR “ASD”) AND (“gut microbiota” OR “gut microbiome” OR “dysbiosis” OR “fecal microbiota transplantation” OR “probiotics” OR “prebiotics”). We included original research articles (cross-sectional, cohort, case–control, randomized controlled trials), meta-analyses, and animal studies that reported gut microbiota composition, metabolic or immune parameters, or intervention outcomes in ASD. Studies were excluded if they lacked a neurotypical control group (for observational studies), had sample size < 10 per group, or were case reports, conference abstracts, or non-English publications. The final reference list was curated to include both foundational prior reviews and the most recent advances within the 10-year window. Therefore, this review integrates findings from studies to provide a comprehensive overview of ASD-associated gut microbiota alterations, underlying mechanisms, and emerging therapeutic strategies. In addition, prenatal and postnatal environmental factors also contribute to ASD susceptibility. Maternal nutrition (e.g., vitamin D deficiency [VDD]), exposure to pollutants or endocrine disruptors, prenatal stress, and metabolic conditions such as gestational diabetes have been associated with altered offspring gut microbiota and increased ASD risk. Early-life factors including breastfeeding, antibiotic use, and caregiving practices further shape the developing gut-brain axis. These influences are discussed where relevant. We also discuss the role of the gut virome and mycobiome, host genetics, epigenetics, and diagnostic biomarker development.

## 2 Gut microbiota composition in ASD: consistent patterns and heterogeneity

### 2.1 Diversity and phylum-level changes

The pathophysiology of ASD is multifactorial, involving genetic mutations (e.g., in synaptic scaffolding proteins, ion channels, and neurodevelopmental genes), environmental factors (e.g., prenatal infection, maternal immune activation, VDD), and their interactions. Neuroinflammation, oxidative stress, and mitochondrial dysfunction are consistently observed in ASD brains, alongside alterations in neurotransmitter systems (glutamate/GABA, serotonin, dopamine). Importantly, many of these pathological processes are influenced by gut microbiota via the gut-brain axis.

The assessment of gut microbial diversity has yielded complex and partly inconsistent findings across ASD studies, yet a coherent picture emerges when considering both α-diversity (within-sample richness) and β-diversity (between-sample compositional differences). Most meta-analyses and large cohort studies indicate that while α-diversity may be unchanged or only modestly reduced in ASD compared to TD controls (Ding et al., 2021; Xie et al., 2022), the β-diversity almost invariably separates ASD from TD groups, suggesting that the overall microbial community structure is distinctly altered in ASD (Wan et al., 2022; Xie et al., 2022; Zeng et al., 2025). For instance, a large Chinese study by Wan et al. (2022) paradoxically found increased bacterial richness in ASD children, whereas other studies reported no difference in Chao1 or ACE indices (Ding et al., 2021; Wan et al., 2022). This variability likely reflects differences in age range, dietary habits, geographic location, and antibiotic exposure across cohorts. Notably, the systematic review by Jurek et al. (2021) concluded that despite methodological heterogeneity, 28 out of 31 studies reported some form of dysbiosis, underscoring the robustness of the overall finding (Jurek et al., 2021).

At the phylum level, a commonly observed but not universal alteration is an increased abundance of *Bacteroidetes* and a correspondingly decreased *Firmicutes/Bacteroidetes* (F/B) ratio in children with ASD, a pattern observed across multiple independent studies and meta-analyses (Iglesias-Vázquez et al., 2020; Kadiyska et al., 2025; Zhang et al., 2018; Zou et al., 2020). A recent large Bulgarian study (Kadiyska et al., 2025), which included 302 ASD children and adolescents, confirmed significantly lower F/B ratios, with the youngest age group (0–4 years) exhibiting the most pronounced deviations (Kadiyska et al., 2025). Similarly, Zou et al. (2020) reported a significantly higher *Bacteroidetes/Firmicutes* ratio in Chinese ASD children due to enrichment of *Bacteroidetes* (Zou et al., 2020), and Zhang et al. (2018) replicated this finding in another Chinese cohort (Zhang et al., 2018). However, not all studies agree on the direction of change at the phylum level. Some have reported no significant differences in dominant phyla between ASD and TD groups (De Sales-Millán et al., 2024), while others have found increases in *Actinobacteria* and *Proteobacteria* (Xie et al., 2022). For example, Xie et al. (2022) observed significantly higher relative abundances of *Actinobacteria* and *Proteobacteria* in 101 Chinese ASD children compared to controls (Xie et al., 2022). Additionally, Ding et al. (2021) noted notable differences in *Actinobacteria* and *Firmicutes* between groups (Ding et al., 2021), and Fujishiro et al. (2023) found an increased abundance of *Firmicutes* specifically in preterm-born children with ASD (Fujishiro et al., 2023). These discrepancies can be partly reconciled by considering geographic and dietary factors: Chinese cohorts tend to show higher *Proteobacteria* and lower *Firmicutes*, whereas European cohorts more consistently highlight *Bacteroidetes* elevations. Furthermore, the presence of GI symptoms, which are more frequent in severe ASD, may modulate phylum-level profiles. For instance, Dan et al. (2020) reported that ASD children with chronic constipation exhibited depletion of *Bacteroides* and *Prevotella*, contrasting with the general pattern of *Bacteroidetes* increase (Dan et al., 2020). Biologically, a lower F/B ratio may reflect a shift in short-chain fatty acid (SCFA) production profiles: Bacteroidetes are predominantly associated with acetate and propionate, whereas Firmicutes are major butyrate producers. Reduced butyrate has been linked to impaired gut barrier function, increased intestinal permeability, and systemic inflammation, providing a plausible mechanistic link between phylum-level changes and ASD pathophysiology. However, the F/B ratio is an aggregate measure that can obscure important genus- and species-level alterations, and its interpretation is highly dependent on age, diet, and geographic location. Therefore, this metric should be viewed as one descriptive parameter among many rather than a standalone diagnostic or mechanistic marker. Thus, while a decreased F/B ratio due to relative *Bacteroidetes* enrichment is a common theme, the specific phylum-level signature is modulated by age, geography, diet, GI comorbidity, and possibly by the sequencing platform and variable region of the 16S rRNA gene used. Taken together, the evidence supports that gut microbial community composition at the phylum level is significantly perturbed in ASD, with the F/B ratio emerging as a potential, though not universal, biomarker of dysbiosis.

### 2.2 Genus- and species-level signatures

Moving from phylum-level alterations to finer taxonomic resolution, meta-analyses of 16S rRNA sequencing studies have reproducibly identified several bacterial genera that differ in relative abundance between children with ASD and neurotypical controls. Among the most consistent findings are a decreased abundance of *Bifidobacterium* and increased abundances of *Clostridium*, *Bacteroides*, *Faecalibacterium*, and *Parabacteroides* in the ASD group (Iglesias-Vázquez et al., 2020; Lin et al., 2024; Yang et al., 2024). The meta-analysis by Iglesias-Vázquez et al. (2020), which synthesized data from 18 studies, quantified these differences by demonstrating significantly higher relative abundances of *Bacteroides*, *Parabacteroides*, *Clostridium*, *Faecalibacterium*, and *Phascolarctobacterium*, alongside lower abundances of *Coprococcus* and *Bifidobacterium*, in children with ASD compared to controls (Iglesias-Vázquez et al., 2020). A subsequent and larger meta-analysis by Yang et al. (2024), encompassing 28 studies, extended this taxonomic inventory by reporting elevated levels of *Anaerostipes*, *Dorea*, *Lachnoclostridium, Catenibacterium*, and *Collinsella*, as well as reduced levels of *Barnesiella*, *Odoribacter*, *Paraprevotella*, Turicibacter, *Lachnospira*, *Pseudomonas*, *Parasutterella*, and *Haemophilus* in ASD (Yang et al., 2024). Notably, discrepancies in the direction of change for certain genera, such as *Faecalibacterium*, which some individual studies reported as increased and others as decreased, tended to converge after meta-analytic pooling, suggesting that small sample sizes and methodological variability may obscure true effects in isolated reports (Yang et al., 2024).

At the species level, a growing body of evidence has pinpointed specific microbial taxa with potential functional relevance. A comprehensive meta-analysis by Lin et al. (2024), which integrated 43 studies, confirmed lower abundances of *Bifidobacterium* (genus) and *Parabacteroides*, and higher abundances of *Bacteroides* and *Clostridium* (genus) in ASD (Lin et al., 2024). Species-specific alterations have been further delineated: decreased abundances of *Faecalibacterium prausnitzii*, *Prevotella copri, Bacteroides fragili*s, and *Akkermansia muciniphil*a are frequently observed in ASD (Bojović et al., 2020; Retuerto et al., 2024), whereas increased abundances of *Bacteroides vulgatus* and *Prevotella copri* have also been reported (Zou et al., 2020). The consistent depletion of *Akkermansia muciniphila* is of particular interest, given its role as a mucin-degrading bacterium that supports gut barrier integrity and exerts anti-inflammatory effects through butyrate production (Goo et al., 2020; Li et al., 2024; Retuerto et al., 2024; Wang et al., 2025). Similarly, reduced *Bifidobacterium longum* has been documented in multiple ASD cohorts, and its abundance has been shown to increase following successful probiotic interventions, suggesting a potential therapeutic target (Wang et al., 2020).

Geographic and population-specific heterogeneity, however, modulates the expression of these microbial signatures. In Chinese cohorts, the most pronounced changes include an increase in *Escherichia-Shigella* and decreases in *Blautia* and *Lachnospiraceae* (Wan et al., 2022; Xie et al., 2022). For example, Xie et al. (2022) analyzed 101 children with ASD and found significantly higher relative abundance of *Escherichia-Shigella* and significantly lower relative abundances of *Blautia* and unclassified *Lachnospiraceae* compared to neurotypical controls (Xie et al., 2022). In contrast, Indian children with ASD exhibited higher abundances of *Lactobacillaceae* and *Bifidobacteriaceae* (Pulikkan et al., 2018), while Egyptian children showed increased *Bacteroides* and *Ruminococcus* together with decreased *Bifidobacterium* (Ahmed et al., 2020). A family-based study by Chen et al. (2025) further confirmed that children with ASD had lower *Bifidobacterium* and higher *Bacteroides* and *Clostridium* than their non-ASD siblings, indicating that shared familial factors—including genetics, diet, and household environment—contribute to these microbial traits (Chen et al., 2025). Taken together, these geographic and familial variations underscore the importance of context-specific reference ranges when considering microbial biomarkers for ASD.

Beyond the genera consistently identified in meta-analyses, several other taxa have been reported in individual studies, awaiting confirmation in larger, independent cohorts. Ma et al. (2019) observed lower abundances of *Lachnoclostridium*, *Tyzzerella*, *Flavonifractor*, and unidentified *Lachnospiraceae* in in 45 Chinese children with ASD and 45 matched neurotypical controls (Ma et al., 2019). Toscano de Oliveira et al. (2025) found enrichment of *Clostridium sensu stricto 1*, the *Ruminococcus* torques group, and *Bifidobacterium* breve in ASD (10 male ASD children, mean age 6.2 years; 10 male neurotypical controls, mean age 6.1 years) (Toscano de Oliveira et al., 2025). Zeng et al. (2025) demonstrated that children with severe ASD exhibited a distinct microbiota composition from those with mild ASD (total 46 participants across neurotypical, mild ASD, and severe ASD groups), with the latter showing greater similarity to neurotypical children, suggesting a gradient of dysbiosis that correlates with symptom severity (Zeng et al., 2025). Guo et al. (2025) reported decreased *Bifidobacterium* and *Veillonella* alongside increased Streptococcus in children with poor neurodevelopmental outcomes (338 children aged 0-3 years: 169 poor neurodevelopment, 169 normal neurodevelopment), further linking microbial imbalances to neurocognitive function (Guo et al., 2025). Collectively, these findings converge on a consistent theme: a reduction in putatively beneficial, anti-inflammatory commensals—particularly *Bifidobacterium* and *Akkermansia muciniphila*—and an expansion of potentially pro-inflammatory or pathogenic taxa, including *Clostridium*, *Bacteroides*, and *Escherichia-Shigella*, in the ASD gut microbiome. This compositional imbalance is hypothesized to contribute to the chronic low-grade inflammation and impaired intestinal barrier function observed in a substantial proportion of affected children, thereby providing a mechanistic link between gut dysbiosis and ASD pathophysiology (Table 1).

**TABLE 1:** Consistent gut microbiota alterations in children with ASD at different taxonomic levels.

Taxonomic level	Direction in ASD	Representative taxa	Key notes/heterogeneity	References
Phylum	↑	*Bacteroidetes*	Most consistent finding: decreased Firmicutes/Bacteroidetes ratio; some studies report increased Proteobacteria or Actinobacteria (e.g., in Chinese cohorts)	(Iglesias-Vázquez et al., 2020; Kadiyska et al., 2025; Zhang et al., 2018; Zou et al., 2020)
	↓	Firmicutes (relative abundance)		(Iglesias-Vázquez et al., 2020; Kadiyska et al., 2025; Zhang et al., 2018; Zou et al., 2020)
Genus	↑	*Clostridium*, *Bacteroides*, *Faecalibacterium*, *Parabacteroides*, *Escherichia-Shigella* (China)	Geographic variability: *Escherichia-Shigella* increase prominent in China; *Lactobacillus* increase reported in India	(Iglesias-Vázquez et al., 2020; Lin et al., 2024; Yang et al., 2024)
	↓	*Bifidobacterium*, *Blautia*, *Akkermansia*, *Coprococcus*	Reduced *Bifidobacterium* among the most reproducible findings across meta-analyses	(Iglesias-Vázquez et al., 2020; Lin et al., 2024; Pulikkan et al., 2018; Yang et al., 2024)
Species	↑	*Bacteroides vulgatus*	*Prevotella copri* reported as both increased and decreased in different studies	(Zou et al., 2020)
	↓	*Faecalibacterium prausnitzii*, *Akkermansia muciniphila*, *Bacteroides fragilis*, *Bifidobacterium longum*	Depletion of *A. muciniphila* linked to gut barrier dysfunction	(Bojović et al., 2020; Goo et al., 2020; Li et al., 2024; Retuerto et al., 2024; Wang et al., 2025)
Modifiers	Age: most pronounced dysbiosis in children 0–4 years; delayed microbial maturation in ASD	Sex: *Megamonas* exclusively in females with ASD; sex-specific mucosal associations	Geography: Chinese cohorts show *Escherichia-Shigella* ↑ and *Blautia* ↓; European cohorts emphasize *Bacteroides* ↑	(De Sales-Millán et al., 2024; Kadiyska et al., 2025; Reeves et al., 2024; Tamana et al., 2021; Wan et al., 2022)

ASD, Autism Spectrum Disorder.

### 2.3 Age-, sex-, and geography-specific differences

The gut microbiota is a dynamic ecosystem that undergoes substantial remodeling during childhood, and mounting evidence indicates that the nature and magnitude of ASD-associated dysbiosis are modulated by age, sex, and geographic origin. Regarding age, typically developing children exhibit a progressive increase in gut microbial α-diversity as they mature, reflecting ecological succession and functional specialization of the intestinal microbiome. In contrast, children with ASD fail to display this normative trajectory. Dan et al. (2020) reported that while α-diversity in the control group increased with age, no such change was observed in the ASD group, suggesting a developmental arrest or delay in microbial community maturation (Dan et al., 2020). Wan et al. (2022) extended this observation by demonstrating that the developing dynamics of growth-associated gut bacteria—species that normally increase in abundance with chronological age in healthy children—were absent in ASD across the early-life age spectrum (Wan et al., 2022). The most pronounced deviations appear to occur in the youngest children. A large Bulgarian study by Kadiyska et al. (2025), which included 302 children and adolescents with ASD, found that the 0–4 year age group exhibited the greatest reductions in the F/B ratio and the *Actinobacteria/Proteobacteria* ratio compared to neurotypical reference values, with these differences attenuating in older age groups (Kadiyska et al., 2025). This age-dependent pattern implies that early childhood may represent a critical window during which gut dysbiosis is most pronounced and potentially most amenable to therapeutic intervention.

Sex differences in ASD-associated gut microbiota have received far less attention, yet available evidence points to meaningful sex-specific signatures that cannot be ignored. The male predominance in ASD (approximately 4:1) has historically led to female underrepresentation in microbiome studies, but recent investigations that deliberately include both sexes have revealed distinct patterns. De Sales-Millán et al. (2024) compared gut microbiota composition in male and female children with ASD and neurotypical controls, identifying three genera—*Megamonas, Oscillibacter*, and *Acidaminococcus*—that varied between sexes in both ASD and control groups. Notably, *Megamonas* was exclusively present in females with ASD, suggesting a potential sex-specific microbial marker (De Sales-Millán et al., 2024). Similarly, Reeves et al. (2024) examined mucosa-associated microbiota along the entire GI tract (antrum, duodenum, ileum, right colon, and rectum) and reported sex-specific differences in bacterial composition at multiple anatomical sites, indicating that the influence of sex on gut microbiota is not merely a luminal phenomenon but extends to tissue-associated communities (Reeves et al., 2024). Furthermore, Tamana et al. (2021) demonstrated that a *Bacteroidetes*-dominant gut microbiota at 12 months of age was associated with enhanced cognitive and language development at 2 years, but this association was significant only in male infants, not in females (Tamana et al., 2021). These sex-dependent effects underscore the necessity of stratifying analyses by sex in future studies, as pooling sexes may obscure important biological distinctions and lead to incorrect generalizations.

Geographic variation represents a third major source of heterogeneity in ASD microbiome research, reflecting differences in diet, lifestyle, antibiotic use, and genetic background across populations. Chinese cohorts consistently exhibit a microbial profile that differs from European and North American cohorts. For instance, Wan et al. (2022) and Xie et al. (2022) independently reported increased relative abundance of *Escherichia-Shigella* and decreased *Blautia* and *Lachnospiraceae* in Chinese children with ASD (Wan et al., 2022; Xie et al., 2022). This pattern contrasts with European studies, where elevations in *Bacteroides* and *Clostridium* are more commonly emphasized (Iglesias-Vázquez et al., 2020; Kadiyska et al., 2025). In the Indian population, Pulikkan et al. (2018) found higher abundances of *Lactobacillaceae* and *Bifidobacteriaceae* in children with ASD compared to family-matched controls (Pulikkan et al., 2018), a finding not consistently replicated in other geographic regions. Egyptian children with ASD exhibited increased *Bacteroides* and *Ruminococcus* alongside decreased *Bifidobacterium* (Ahmed et al., 2020), partially overlapping with but also distinct from Chinese and European profiles. Similarly, a Mexican family study reported that the gut microbiome of children with ASD more closely resembled that of their mothers than their fathers, suggesting a maternal transmission or shared environmental effect (Mendoza et al., 2026). Collectively, these geographic and familial observations caution against the search for a universal microbial signature of ASD. Instead, they argue for the development of population-specific reference databases and for the inclusion of geographically diverse cohorts in future meta-analyses to disentangle true disease-associated signals from background environmental variation.

### 2.4 Gut microbiota in specific ASD subgroups

Beyond group-level comparisons between ASD and neurotypical populations, a growing body of research has examined gut microbiota profiles within clinically defined ASD subgroups, revealing that the presence of GI comorbidity, preterm birth, or co-occurring neurological impairments substantially modifies the microbial signature. Children with ASD who experience chronic constipation—one of the most common GI complaints—exhibit a distinct dysbiotic pattern compared to ASD children without significant GI symptoms. Dan et al. (2020) specifically investigated a subgroup of ASD children with chronic constipation (C-ASD) and found that, relative to age-matched typically developing controls, the C-ASD group displayed decreased microbial diversity, depletion of Sutterella, Prevotella, and Bacteroides species, and dysregulation of associated metabolic pathways, including those involved in neurotransmitter metabolism (serotonin, dopamine, histidine, and GABA) (Dan et al., 2020). Importantly, the severity of GI symptoms has been shown to correlate with the degree of microbial alteration and with ASD symptom severity itself. Ding et al. (2021) reported that children with ASD who had high GI severity scores exhibited significantly higher Autism Treatment Evaluation Checklist scores than those with low GI scores, establishing a dose-dependent relationship between GI dysbiosis and behavioral manifestations (Ding et al., 2021). Similar positive correlations between GI dysfunction and behavioral impairments have been reported by other studies (Adams et al., 2011; Chaidez et al., 2014; Gorrindo et al., 2012).

Preterm birth represents another important modifying factor, as it is associated with both an increased risk of ASD and a distinctive pattern of gut microbiota development. Two studies focusing on preterm-born children with ASD have provided complementary insights. Fujishiro et al. (2023) compared preterm-born children with ASD to preterm-born typically developing children and found that the ASD group exhibited significantly higher α-diversity and a greater relative abundance of the phylum Firmicutes (Fujishiro et al., 2023). This finding is notable because, in full-term populations, ASD is often associated with unchanged or reduced α-diversity; the opposite direction in preterm infants suggests that the interplay between prematurity and ASD risk produces a unique microbial ecology. Extending this line of inquiry, Rozé et al. (2020) conducted a large prospective cohort study of very preterm newborns (gestational age < 32 weeks) and identified five discrete gut microbiota clusters at 4 weeks of postnatal age. Clusters characterized by *Enterococcus*, *Staphylococcus*, or low bacterial load (non-amplifiable samples) were significantly associated with an increased risk of death or neurodevelopmental delay at 2 years of corrected age, even after adjusting for confounders such as gestational age and neonatal intensive care unit practices (Rozé et al., 2020). More recently, Tian et al. (2026) employed a prospective, matched, longitudinal multi-omics design in 60 preterm infants, classifying them at 3 months corrected age as high-risk or low-risk for neurodevelopmental impairment based on combined motor and neurological assessments. The high-risk trajectory was characterized by dominance of *Klebsiella variicola* and enrichment of pathways related to bacterial virulence, stress response, and human neurodegenerative disease pathways, whereas the low-risk group showed enrichment of *Akkermansia muciniphila* and core biosynthetic pathways (Tian et al., 2026). Notably, meconium features already correlated with 3-month neurobehavioral scores, suggesting that microbial biomarkers of neurodevelopmental risk may be detectable from birth. In a related but distinct patient population, Fundora et al. (2026) examined infants with congenital heart disease, a condition associated with both gut dysbiosis and neurodevelopmental delays. They found that lower cognitive and language scores were associated with lower abundances of *Parabacteroides*, *Bacteroides*, and *Bifidobacterium*, while lower motor scores correlated with higher abundances of pro-inflammatory genera *Serratia*, *Acinetobacter*, and *Proteus* (Fundora et al., 2026). These findings underscore the general principle that early-life gut dysbiosis—regardless of the specific underlying medical condition—may contribute to adverse neurodevelopmental outcomes through shared inflammatory and metabolic pathways.

Finally, children with severe motor and intellectual disabilities (SMID) who receive long-term enteral nutrition represent an extreme phenotype of gut dysbiosis that offers insights into the combined effects of limited dietary diversity and neurological impairment. Nakai et al. (2023) analyzed fecal samples from 10 children with SMIDs receiving enteral nutrition via gastric fistula or tube and compared them to 19 healthy controls. The SMID group exhibited significantly lower α-diversity, reduced abundances of Clostridiales and butyrate-producing bacteria, and increased abundances of *Bacteroidales*. Dietary assessment revealed that fiber intake in the SMID group was approximately two-thirds of the estimated average requirement for healthy Japanese children, suggesting that inadequate prebiotic substrate contributes to the loss of beneficial fermentative bacteria (Nakai et al., 2023). Although not all children with ASD receive enteral nutrition, this subgroup illustrates how severe neurological compromise coupled with dietary restrictions can exacerbate dysbiosis, potentially creating a vicious cycle of gut dysfunction and behavioral deterioration. Taken together, these subgroup analyses demonstrate that the gut microbiota profile in ASD is not monolithic; rather, it is shaped by the presence of GI comorbidity, gestational age at birth, co-occurring medical conditions, and nutritional status. Recognizing this heterogeneity is essential for designing targeted microbiome-based interventions that address the specific needs of distinct ASD subtypes.

## 3 The gut virome and mycobiome in ASD

### 3.1 Gut virome

While the vast majority of research on the gut microbiome in ASD has focused on bacterial communities, emerging evidence indicates that non-bacterial microbial components—specifically the gut virome (the collective community of viruses, including bacteriophages) and the gut mycobiome (the fungal community)—are also altered in ASD and may contribute to disease pathophysiology through mechanisms distinct from those of bacteria. The gut virome is dominated by bacteriophages that infect and lyse bacterial hosts, thereby influencing bacterial community structure, horizontal gene transfer, and microbial metabolic output. Wan et al. (2024) performed a metagenomic analysis of gut DNA viruses in 60 children with ASD and 64 age- and sex-matched typically developing controls, revealing that ASD was associated with a significantly altered virome composition characterized by enrichment of *Clostridium* phage, *Bacillus* phage, and *Enterobacteria* phage (Wan et al., 2024). Importantly, the authors demonstrated that these ASD-enriched phages were associated with disrupted viral ecology—specifically, reduced viral-bacterial interaction network complexity—and that changes in bacteriome-virome interplay influenced the microbial potential for biosynthesis of neuroactive metabolites, including GABA and tryptophan derivatives. This suggests that phages do not merely reflect bacterial hosts but may actively modulate the functional capacity of the residual bacterial community. In a previous open-label trial of microbiota transfer therapy (MTT), Kang et al. (2017) also observed that successful engraftment of donor bacteria was accompanied by shifts in bacteriophage populations, implying that phage dynamics are an integral component of therapeutic microbiome manipulation (Kang et al., 2017).

### 3.2 Gut mycobiome

Parallel to these virome alterations, the gut mycobiome of children with ASD exhibits distinct compositional changes that may contribute to GI and systemic inflammation. Zou et al. (2021) characterized the fungal microbiota in 48 children with ASD and 48 healthy controls using 16S and ITS sequencing, reporting that at the genus level, *Saccharomyces* and *Aspergillu*s showed the most pronounced differences: *Saccharomyces* accounted for 59.07% of fungal genera in ASD versus 40.36% in controls. At the species level, *Aspergillus versicolor* was decreased, whereas *Saccharomyces cerevisiae* was increased in children with ASD relative to controls (Zou et al., 2021). These findings suggest that fungal dysbiosis occurs alongside bacterial changes in ASD; however, whether it is independent of or secondary to bacterial dysbiosis remains to be determined. Retuerto et al. (2024) extended these observations by comparing the gut bacteriome and mycobiome of ASD subjects with their non-ASD siblings, thereby controlling for shared genetic and environmental factors. They reported a significant increase in the fungal phylum *Ascomycota* (98.3% in ASD vs. 94% in siblings) and a trend toward increased *Candida albicans* (27.1% vs. 13.2%) in ASD (Retuerto et al., 2024). The elevation of *Candida albicans* is particularly noteworthy, as this opportunistic yeast can promote inflammation, produce immunomodulatory metabolites, and form biofilms that disrupt the intestinal epithelial barrier.

Further evidence for the clinical relevance of fungal dysbiosis comes from a culture-based study by Iovene et al. (2017), who isolated *Candida* spp. from stool samples of 57.5% of ASD subjects compared to 0% of controls. Notably, microscopic examination revealed the presence of pseudo-hyphae in *Candida isolates* from ASD subjects—a morphological form associated with enhanced adhesion to intestinal mucosa and invasive potential (Iovene et al., 2017). The same study also documented low-grade intestinal inflammation (elevated fecal calprotectin) and increased intestinal permeability in ASD subjects, with a significant linear correlation between disease severity (measured by the Childhood Autism Rating Scale, CARS) and calprotectin levels. This triad of fungal overgrowth, mucosal adherence, and inflammation suggests a mechanistic pathway whereby gut fungi may contribute to the gut-brain axis dysfunction in ASD. More recently, Nirmalkar et al. (2024) reported elevated *Candida albicans* in children with ASD and further explored its potential metabolic contributions, suggesting that *C. albicans* overgrowth may influence tryptophan metabolism and contribute to neuroinflammation (Nirmalkar et al., 2024). Collectively, the virome and mycobiome findings underscore that the gut ecosystem in ASD is perturbed across multiple microbial kingdoms. Bacteriophages likely influence bacterial community function and metabolic output, while fungal dysbiosis may directly promote intestinal inflammation and barrier dysfunction. Future studies should integrate virome and mycobiome profiling alongside bacterial metagenomics to obtain a complete picture of host-microbe interactions in ASD and to identify novel therapeutic targets, such as phage-based modulation of pathobionts or antifungal strategies for subsets of patients with documented Candida overgrowth.

## 4 Gut microbial metabolites, immune dysregulation, and host factors in ASD

### 4.1 SCFAs and gut barrier integrity

**FIGURE 1:**
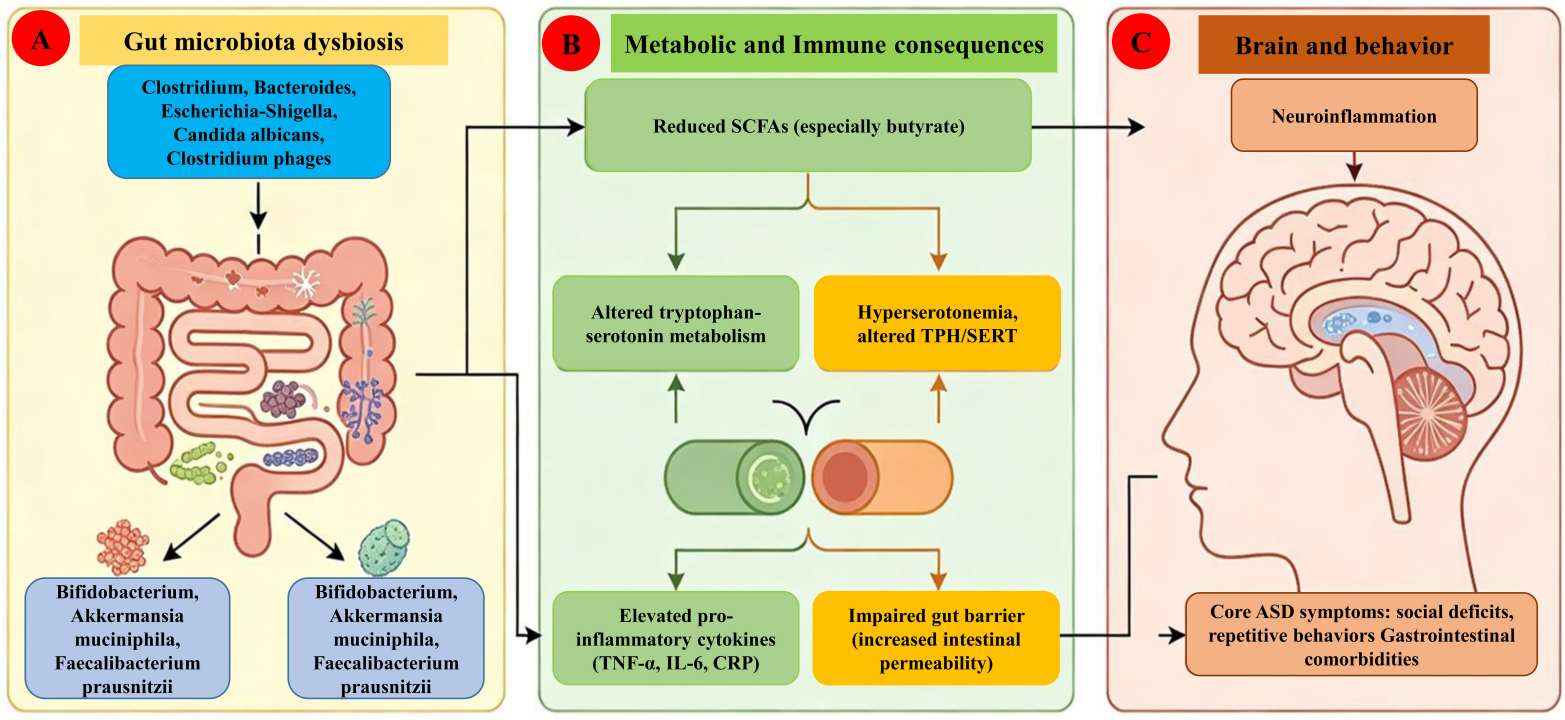
Schematic illustration of gut microbiota dysbiosis, metabolic/immune alterations, and gut–brain axis dysfunction in autism spectrum disorder (ASD). **(A)** Gut microbiota composition changes in ASD: increased levels of *Clostridium*, *Bacteroides*, *Escherichia-Shigella*, *Candida albicans*, and *Clostridium phages*, together with decreased levels of *Bifidobacterium*, *Akkermansia muciniphila*, and *Faecalibacterium prausnitzii.* **(B)** Consequent metabolic and immune alterations: reduced short-chain fatty acids (especially butyrate), disrupted tryptophan-serotonin metabolism (hyperserotonemia, altered TPH/SERT expression), elevated pro-inflammatory cytokines (TNF-α, IL-6, CRP), and impaired gut barrier integrity. **(C)** Effects on the central nervous system via the gut–brain axis: neuroinflammation, synaptic dysfunction, and manifestation of core ASD symptoms (social deficits, repetitive behaviors) as well as gastrointestinal comorbidities. Arrows indicate proposed causal pathways.

SCFAs, primarily acetate, propionate, and butyrate, are key microbial metabolites that influence host physiology through multiple mechanisms, including energy metabolism, gut barrier function, immune regulation, and neurodevelopment. Several studies have reported altered SCFA profiles in children with ASD. Wang et al. (2020) found significantly lower levels of acetic acid, propionic acid, and butyric acid in the feces of children with ASD compared to neurotypical controls, and these levels increased toward control values after probiotic plus fructo-oligosaccharide intervention (Wang et al., 2020). Bojović et al. (2020) examined children with various neurodevelopmental disorders, including ASD. Compared to healthy controls, the neurodevelopmental disorder group had fewer butyrate-producing bacteria (*Faecalibacterium prausnitzii, Butyricicoccus pullicaecorum, Eubacterium rectale)*, and the positive correlation between bacterial abundance and fecal SCFA levels seen in controls was absent in the neurodevelopmental disorder group (Bojović et al., 2020). This functional impairment parallels findings from ASD-only studies, where reduced butyrate is consistently reported, suggesting that butyrate deficiency may be a shared feature across neurodevelopmental disorders that is particularly relevant to ASD pathophysiology (Bojović et al., 2020). In a valproate-induced autistic-like rat model, Kong Q. et al. (2021) reported decreased total SCFAs and butyric acid, alongside increased propionic acid, in feces of autistic-like rats. Notably, correlations between autistic-like behaviors and butyric and propionic acid levels remained consistent regardless of diet or age, indicating that these SCFA alterations may be directly linked to behavioral phenotypes (Kong Q. et al., 2021). The modified ketogenic diet (KD), which increased butyrate kinase gene expression in the gut, also reduced pro-inflammatory cytokines and improved sociability in a pilot study of children with ASD (Allan et al., 2024). Collectively, these findings support the hypothesis that reduced butyrate production and an elevated propionate/butyrate ratio contribute to gut barrier dysfunction, systemic inflammation, and neurobehavioral abnormalities in ASD (Figure 1).

Mechanistically, butyrate serves as the primary energy source for colonocytes and strengthens intestinal barrier function by upregulating tight junction proteins (e.g., claudin-1, occludin, ZO-1). Reduced butyrate production leads to increased intestinal permeability, allowing bacterial lipopolysaccharides and other pro-inflammatory molecules to enter the circulation. This systemic translocation triggers immune activation and low-grade inflammation, which can affect the brain via the vagus nerve and circulating cytokines. Acetate and propionate also modulate immune responses and gut hormone secretion, but butyrate deficiency is considered the most critical for gut barrier dysfunction.

### 4.2 Tryptophan-serotonin metabolism and neurotransmitter pathways

Tryptophan is an essential amino acid that serves as the precursor for serotonin, a neurotransmitter critically involved in mood, behavior, sleep, and GI motility. Dysregulation of tryptophan-serotonin metabolism has been consistently observed in ASD. Xiao et al. (2021) demonstrated that germ-free mice colonized with ASD child microbiota (ASD-FMT mice) exhibited altered tryptophan and serotonin metabolism in both the colon and the prefrontal cortex, with changes in the expression of tryptophan hydroxylase (TPH1, TPH2), serotonin transporter (SERT), and serotonin 1A receptor (5-HT1AR). These metabolic changes were associated with ASD-like behaviors, and similar alterations were detected in the fecal samples of the human ASD donors (Xiao et al., 2021). Dan et al. (2020) analyzed fecal metabolites in children with ASD and chronic constipation, revealing that differential metabolites were enriched in pathways involving serotonin, dopamine, histidine, and GABA, and that these metabolite differences correlated with the altered abundances of specific bacteria (Dan et al., 2020). Wang et al. (2020) reported that children with ASD exhibited a hyperserotonergic state (elevated plasma serotonin) and a dopamine metabolism disorder (decreased homovanillic acid), and that probiotic + fructo-oligosaccharides (FOS) intervention partially normalized both abnormalities while also improving clinical symptoms (Wang et al., 2020). Beyond serotonin and dopamine, other neurotransmitter-related metabolites have been implicated. In a multi-omics study of neurodevelopmental disorders, Wang et al. (2025) identified 29 common metabolites in feces and plasma that were differentially regulated, including *L-asparagine*, *sarcosine*, and *trigonelline*, with integrated analysis linking these metabolites to depleted species such as *Akkermansia muciniphila* and *Lactococcus lactis* (Wang et al., 2025). Although this study included a mixed neurodevelopmental disorder population (ASD and other diagnoses), the observed depletion of *A. muciniphila* and the associated metabolomic shifts are consistent with findings from ASD-specific cohorts, reinforcing the relevance of these microbial-metabolic axes to ASD pathophysiology. These converging lines of evidence indicate that gut microbiota dysbiosis in ASD leads to widespread disturbances in neurotransmitter metabolism, which may directly influence brain function via the gut-brain axis (Figure 1).

### 4.3 Other metabolites and integrated multi-omics signatures

The application of untargeted metabolomics and multi-omics integration has substantially expanded the list of metabolites altered in ASD beyond SCFAs and tryptophan derivatives. Kang et al. (2020) measured 619 plasma metabolites in children with ASD before and after MTT and found that eight metabolites—including nicotinamide riboside, iminodiacetate, methylsuccinate, galactonate, and sarcosine—were significantly lower in the ASD group at baseline, while caprylate and heptanoate were higher. MTT drove global shifts in plasma profiles, notably affecting nicotinate/nicotinamide and purine metabolism (Kang et al., 2020). In the same cohort, p-cresol sulfate was relatively higher in ASD at baseline, and its levels decreased after MTT, inversely correlating with Desulfovibrio abundance (Kang et al., 2020). The potential of urinary organic acids as biomarkers was examined by Daneberga et al. (2022), who found that while p-cresol and 3-(3-hydroxyphenyl)-3-hydroxypropionic acid (HPHPA) were elevated in some ASD children, HPHPA was not a reliable specific marker for Clostridium overgrowth; increased Firmicutes abundance alone might contribute to elevated p-cresol (Daneberga et al., 2022). A large integrated metagenomic and metabolomic study by Chen et al. (2026) across over 1,100 children identified 35 species, 213 genes, 28 pathways, and 99 metabolites associated with ASD. Furthermore, 1,369 single-nucleotide variants, 233 insertions/deletions, and 195 structural variants exhibited differential abundance. Integrated analysis uncovered 357 neurological associations, with mediation analysis showing that several metabolites link microbial variants to the ASD phenotype (Chen et al., 2026). Shao et al. (2024) proposed a “TNFα-sphingolipids-steroid hormones” axis based on integrative meta-omics: increased TNFα correlated with upregulation of microbial sphingolipid metabolism, which in turn negatively correlated with steroid hormones such as estriol and deoxycorticosterone, providing a mechanistic link between gut dysbiosis, neuroinflammation, and hormonal dysregulation (Shao et al., 2024). Li et al. (2024) further demonstrated that a combined probiotic and medium-carbohydrate diet intervention modulated fecal metabolites associated with lysine, glutamate, GABA, arachidonic acid, myristic acid, and vitamin B3, supporting the feasibility of metabolome-guided therapeutic monitoring (Li et al., 2024; Figure 1).

### 4.4 Cytokines, inflammation, and the immune response

Chronic low-grade inflammation is a recognized feature of ASD, and gut microbiota dysbiosis is thought to contribute to this inflammatory state through increased intestinal permeability and translocation of bacterial products. A meta-analysis by Lin et al. (2024) of 43 studies found significantly higher levels of C-reactive protein (CRP) and GABA, and lower levels of oxytocin, iron, and zinc, in children with ASD compared to controls (Lin et al., 2024). Pro-inflammatory cytokines, particularly TNF-α and IL-6, have been repeatedly reported to be elevated in ASD. Wang et al. (2022) studied children with attention-deficit/hyperactivity disorder (ADHD) and found lower plasma TNF-α levels in ADHD compared to controls (Wang et al., 2022). This contrasts with the consistently elevated TNF-α reported in ASD, indicating that while both are neurodevelopmental conditions, their peripheral immune profiles differ markedly—a finding that may have diagnostic and therapeutic implications (Wang et al., 2022). In ASD, the correlation between TNF-α and microbial functions was explored by Shao et al. (2024), who observed positive correlations between TNF-α and microbial pathways such as “Bacterial toxins” and “Lysosome” (Shao et al., 2024). Sanctuary et al. (2019) reported that a combination of *Bifidobacterium infantis* and bovine colostrum reduced IL-13 and TNF-α production in some children with ASD, accompanied by clinical improvement (Sanctuary et al., 2019). Allan et al. (2024) found that a KD decreased plasma IL-12p70 and IL-1β in a small ASD cohort (Allan et al., 2024). Iovene et al. (2017) documented elevated fecal calprotectin, a marker of intestinal inflammation, in ASD subjects, with a significant linear correlation between calprotectin levels and disease severity (CARS score) (Iovene et al., 2017). Taken together, these data support a model wherein gut dysbiosis promotes intestinal and systemic inflammation, which may in turn exacerbate behavioral symptoms through neuroinflammatory mechanisms (Figure 1 and Table 2).

**TABLE 2:** Key gut microbial metabolites, immune/inflammatory markers, and host factors associated with ASD.

Category	Biomarker/ molecule	Typical change in ASD	Associated microbial taxa/mechanisms	References
Short-chain fatty acids	Butyrate, acetate, propionate (fecal)	↓ (especially butyrate)	Reduced butyrate-producing bacteria: *Faecalibacterium prausnitzii*, *Butyricicoccus pullicaecorum*	(Bojović et al., 2020; Wang et al., 2020)
Tryptophan-serotonin metabolism	Plasma serotonin	↑ (hyperserotonergic state)	Altered tryptophan hydroxylase (TPH1, TPH2), SERT, 5-HT1AR expression; modulated by ASD-FMT in mice	(Wang et al., 2020; Xiao et al., 2021)
Other neurotransmitters	Dopamine metabolite (homovanillic acid)	↓	Dopamine metabolism disorder; GABA pathway alterations in chronic constipation subgroup	(Dan et al., 2020; Liu et al., 2021; Wang et al., 2020)
Inflammatory cytokines	TNF-α, IL-6, CRP	↑	Positive correlation with microbial pathways (“Bacterial toxins”, “Lysosome”); fecal calprotectin ↑ correlates with CARS score	(Allan et al., 2024; Iovene et al., 2017; Lin et al., 2024; Shao et al., 2024)
Other plasma metabolites	p-Cresol sulfate, nicotinamide riboside, sarcosine	Variable (e.g., p-cresol sulfate ↑ )	Shifted after microbiota transfer therapy (MTT); associated with *Desulfovibrio* abundance	(Kang et al., 2020)
Trace elements/hormones	Oxytocin, iron, zinc	↓	Meta-analysis finding; oxytocin lower in ASD vs. controls	(Lin et al., 2024)

ASD, Autism Spectrum Disorder; CRP, C-reactive protein.

Cytokines such as TNF-α and IL-6 can influence brain function through multiple mechanisms: (i) they can cross the blood-brain barrier via saturable transport systems; (ii) they activate vagal afferent neurons, which signal brainstem nuclei; and (iii) they induce the production of inflammatory mediators by cerebral endothelial cells and perivascular macrophages. Chronic elevation of these cytokines has been associated with impaired synaptic plasticity, altered neurotransmitter metabolism, and behavioral changes in animal models of ASD.

### 4.5 Host genetics, epigenetics, and the gut-microbiome interface

Host genetic variation contributes to the risk of ASD and may also shape the gut microbiota, creating a bidirectional interplay. Liu et al. (2021) performed whole-exome sequencing in 26 ASD children and matched controls, identifying single nucleotide variations (SNVs) enriched in genes associated with innate immune response, protein glycosylation, and retrograde axonal transport. These SNVs correlated with the composition of gut microbiota and a broad spectrum of microbial functions, especially metabolism. Furthermore, the abundance of metabolites involved in neurotransmitter networks was inferred to be causally related to specific SNVs and microbes (Liu et al., 2021). Family-based studies have also highlighted the heritable component of the gut microbiome. Chen et al. (2020) recruited 76 children with ASD and 47 typically developing children, along with their mothers, and found that gut bacteria shared between children and their mothers (e.g., *Lachnospiraceae*, *Ruminococcaceae*, *Collinsella*) were associated with developmental level and social deficits. The proportion of ASD children sharing certain *Lachnospiraceae* ASVs with their mothers was significantly lower than that of controls, and sharing of protective ASVs was linked to less severe social deficits (Chen et al., 2020). Similarly, Chen et al. (2025) analyzed 19 families with a child with ASD, along with non-ASD siblings and parents, and found that children with ASD had lower *Bifidobacteriu*m and higher *Bacteroides* and *Clostridium* than their siblings, indicating that familial (genetic and environmental) factors play a role, but disease-specific alterations also exist (Chen et al., 2025). A Mexican family study further reported that the gut microbiome of offspring with ASD is more similar to the mother’s than the father’s microbiome, suggesting potential maternal transmission or shared early-life exposure (Mendoza et al., 2026).

Beyond genetic variation, epigenetic modifications have emerged as another layer of host-microbiome interaction. Cuomo et al. (2023) performed whole-genome methylation analysis of human fecal DNA from 8 ASD children and 7 healthy controls. They found specific epigenetic signatures in human fecal DNA, especially at genes related to inflammation, that associated with ASD. Using a methylation-based deconvolution algorithm, they showed that human fecal DNA derived mainly from immune cells, and the relative abundance of cell types differed between patients and controls. Notably, both epigenetic age and microbiota age were accelerated in ASD children, suggesting that the gut ecosystem ages prematurely (Cuomo et al., 2023). These findings open new avenues for non-invasive diagnostic biomarkers based on host epigenetic marks in stool. In summary, the gut microbiota in ASD does not operate in isolation; it is intimately linked to host genetic variants, epigenetic modifications, and immune status. Future research should integrate these dimensions to build a system-level understanding of ASD pathogenesis and to identify personalized therapeutic targets (Figure 1).

## 5 Preclinical evidence and therapeutic interventions targeting gut microbiota in ASD

### 5.1 Causal evidence from animal models of FMT

Establishing a causal relationship between gut microbiota dysbiosis and ASD-related behaviors is essential for translating observational findings into therapeutic strategies. While cross-sectional human studies can identify associations, they cannot determine whether microbial alterations are a cause or a consequence of the disorder. Animal models, particularly those involving FMT from ASD donors into germ-free or specific-pathogen-free mice, have provided compelling causal evidence. In a landmark study, Xiao et al. (2021) transplanted fecal microbiota from children with ASD or from TD children into germ-free mice (Xiao et al., 2021). The resulting ASD-FMT mice exhibited core ASD-like behaviors, including reduced social interaction and increased repetitive behaviors, whereas TD-FMT mice did not. Furthermore, ASD-FMT mice showed altered gut microbial community structures and, crucially, dysregulated tryptophan and serotonin metabolism in both the colon and the prefrontal cortex (Xiao et al., 2021). Key proteins involved in serotonin synthesis and transport–TPH1) and 2 (TPH2), SERT, and 5-HT1AR—were differentially expressed in ASD-FMT mice compared to controls (Xiao et al., 2021). These findings directly demonstrate that the ASD gut microbiome can induce behavioral abnormalities and metabolic disturbances in recipient animals. Other mouse studies have further supported the causal role of gut microbiota in ASD-like behaviors. Sharon et al. (2019) colonized germ-free mice with microbiota from ASD or TD human donors and found that ASD-colonized mice showed reduced social behavior and altered brain gene expression (Sharon et al., 2019). Avolio et al. (2022) reported that probiotic treatment ameliorated behavioral abnormalities in a valproate-induced mouse model of ASD, partly through restoring gut barrier function (Avolio et al., 2022). Abuaish et al. (2021) demonstrated that maternal immune activation alters offspring gut microbiota and induces ASD-like behaviors, which were partially reversed by probiotic intervention (Abuaish et al., 2021). Together with the findings of Goo et al. (2020) already discussed, these studies provide convergent evidence that gut microbiota manipulation can modulate ASD-relevant phenotypes in rodents (Goo et al., 2020).

Mechanistically, these behavioral changes in ASD-FMT mice were accompanied by altered tryptophan-serotonin metabolism: TPH1 and TPH2 expression was increased in the colon and decreased in the prefrontal cortex, while SERT and 5-HT1AR were also differentially regulated. These molecular alterations suggest that gut microbiota from ASD children can modulate central neurotransmitter pathways via the gut-brain axis. Similarly, the strain-dependent inflammatory and metabolic shifts observed by Prince et al. (2025) indicate that host genetics interact with microbial signals to produce neurobehavioral outcomes.

The host genetic background modulates the behavioral response to ASD microbiota. Prince et al. (2025) transplanted ASD or TD child microbiota into two different mouse strains, BALB/cAnNCrl (Balb/c) and C57BL/6J (BL/6), after depleting the resident microbiota. Balb/c mice receiving ASD-FMT showed decreased exploratory and social behavior, along with evidence of intestinal, systemic, and central inflammation accompanied by metabolic shifts. In contrast, BL/6 mice exhibited fewer changes, indicating that the effects of ASD-associated dysbiosis are strain-dependent and that host genotype is a critical determinant of susceptibility (Prince et al., 2025). This finding has important implications for preclinical modeling and suggests that genetic variability in humans may similarly influence the impact of gut dysbiosis on ASD symptomatology (Table 3).

**TABLE 3:** Preclinical evidence from animal models demonstrating causal role of gut microbiota in ASD-like behaviors.

Model/ intervention	Microbiota manipulation	Main behavioral/ physiological outcomes	Proposed mechanism(s)	References
Germ-free mice colonized with ASD child microbiota (ASD-FMT)	Fecal microbiota from children with ASD vs. TD controls	ASD-FMT mice: reduced social interaction, increased repetitive behaviors	Altered tryptophan & serotonin metabolism in colon and prefrontal cortex (TPH1, TPH2, SERT, 5-HT1AR)	(Xiao et al., 2021)
Two mouse strains (Balb/c, BL/6) receiving ASD-FMT	FMT from ASD or TD children after microbiota depletion	Balb/c mice: decreased exploratory & social behavior; strain-dependent effects	Intestinal, systemic, and central inflammation + metabolic shifts; host genotype modulates susceptibility	(Prince et al., 2025)
BTBR mouse model (idiopathic ASD-like)	FMT from healthy human donors	Alleviated social deficits, normalized plasma acylcarnitines	Restored vitamin B6 metabolism; vitamin B6 supplementation alone improved social behavior	(Zheng et al., 2024)
Maternal vitamin D deficiency (VDD) mouse model	FMT from control mice to VDD offspring	Reversed ASD-like behaviors, increased cecal SCFAs	Improved colonic tight junction proteins (claudin-1, occludin)	(Cui et al., 2024)
*Fmr1* KO mouse (fragile X syndrome model)	FMT from normal mouse microbiota	Ameliorated memory deficits and social withdrawal	Restored *Akkermansia muciniphila* abundance; reduced brain TNF-α and microglial marker Iba1	(Goo et al., 2020)

ASD, Autism Spectrum Disorder.

### 5.2 Additional animal models of etiological pathways and therapeutic mechanisms

Additional animal models have explored specific etiological pathways and potential therapeutic mechanisms. Zheng et al. (2024) performed FMT from healthy human donors to BTBR mice, an inbred strain that exhibits idiopathic ASD-like traits. FMT treatment alleviated social deficits, normalized the distinct plasma metabolic profile (particularly reducing elevated long-chain acylcarnitines), and linked these phenotypic improvements to specific *Bacteroides* species and vitamin B6 metabolism. Notably, vitamin B6 supplementation alone also improved social behaviors in BTBR mice, suggesting that FMT may act at least in part through restoration of vitamin B6-related metabolic pathways (Zheng et al., 2024). In a maternal VDD model, Cui et al. (2024) showed that offspring of VDD dams during pregnancy and lactation developed ASD-like behaviors and gut dysbiosis. FMT from control mice reversed these behavioral abnormalities, increased cecal SCFA levels, and improved colonic expression of tight junction proteins (claudin-1 and occludin), highlighting the interplay between early nutritional status, gut microbiota, and neurodevelopment (Cui et al., 2024). Finally, Goo et al. (2020) transplanted normal mouse microbiota into Fmr1 knockout mice, a model of fragile X syndrome (the most common monogenic cause of ASD). FMT ameliorated memory deficits and social withdrawal, normalized the abundance of *Akkermansia muciniphila* (which was very low in untreated Fmr1 KO mice), and reduced brain levels of the pro-inflammatory cytokine TNF-α and microglial marker Iba1 (Goo et al., 2020). Collectively, these animal studies establish that gut microbiota alterations are not merely epiphenomena but can causally contribute to ASD-like behaviors, and that correcting dysbiosis through FMT or targeted nutritional interventions can reverse or mitigate these effects.

### 5.3 Clinical trials of FMT in children with ASD

Building on this causal evidence, a growing number of clinical studies have tested whether modulating the gut microbiota can improve GI and behavioral symptoms in children with ASD. The most intensive intervention to date is FMT, often delivered as part of a multi-step protocol. Kang et al. (2017) conducted an open-label trial of MTT in 18 children with ASD, which included a 2-week course of oral vancomycin (to suppress pathogenic bacteria), a bowel cleanse, and then a high initial dose of FMT followed by daily lower maintenance doses for 7–8 weeks (Kang et al., 2017). At the end of treatment, the GI Symptom Rating Scale showed an approximately 80% reduction in GI symptoms, and standardized behavioral assessments (e.g., the Aberrant Behavior Checklist and Social Responsiveness Scale) indicated significant improvements in ASD core symptoms. These benefits persisted at 8-week follow-up. Microbiota analysis revealed successful partial engraftment of donor microbiota, increased overall bacterial diversity, and elevated relative abundances of *Bifidobacterium*, *Prevotella*, and *Desulfovibrio*—taxa commonly depleted in ASD (Kang et al., 2017). A long-term follow-up of the same cohort two years after treatment showed that most GI improvements were maintained, and ASD-related symptoms had improved even further. Bacterial diversity and the abundances of *Bifidobacterium* and *Prevotella* remained significantly elevated compared to baseline, suggesting durable microbial remodeling (Kang et al., 2019). Subsequent shotgun metagenomic analysis of this cohort (Nirmalkar et al., 2022) revealed that MTT increased the relative abundance of beneficial fiber-consuming microbes, including *Prevotella dentalis*, *P. enoeca, P. oris, P. melaninogenica, Bifidobacterium bifidum*, and the sulfur reducer *Desulfovibrio piger.* Importantly, metabolic pathway analysis showed that genes involved in folate biosynthesis, sulfur metabolism, and oxidative stress—which were altered in ASD at baseline—shifted toward levels seen in typically developing children after MTT (Nirmalkar et al., 2022). In a separate study, Liu et al. (2023) used washed microbiota transplantation (WMT) in 24 children with ASD and found that WMT significantly alleviated sleeping disorders and constipation symptoms. The intervention downregulated *Bacteroides*, *Flavonifractor*, and *Parasutterella* while upregulating *Prevotella_9*, and these changes were associated with decreased toxic metabolite production and improved detoxification pathways (e.g., glycolysis, myo-inositol degradation, and fatty acid β-oxidation) (Liu et al., 2023).

More recently, Varnas et al. (2025) conducted a prospective open-label controlled study of colonoscopic FMT in 30 children with ASD (15 FMT, 15 control). At 8 weeks, the intervention group showed significantly greater improvements in the CARS, Parent Global Impression scale, Child Behavior Checklist Internalizing Problems score, and GI symptom rating compared to controls. These improvements persisted up to 18 months for CARS and Parent Global Impression. However, no significant between-group difference was observed for the primary endpoint, change in Autism Diagnostic Observation Schedule (ADOS) scores. No serious adverse events were reported, and three mild, self-limited events occurred (Varnas et al., 2025). The authors caution that the lack of blinding and possible selection bias limit interpretation, underscoring the need for larger, double-blind, placebo-controlled trials (Table 4). Additional FMT studies have reported beneficial effects. Li et al. (2021) conducted an open-label trial of WMT in 40 children with ASD, observing significant improvements in both GI and core ASD symptoms that persisted for 6 months. Wang et al. (2024) performed FMT via nasojejunal tube in 28 children with ASD and found that responders had distinct baseline microbial profiles (higher Prevotella and lower Bacteroides), suggesting the importance of patient stratification (Wang et al., 2024).

**TABLE 4:** Clinical trials of microbiome-based interventions in children with ASD.

Intervention	Study design	N (ASD)	Key findings (GI and/or behavioral)	Microbial changes	Safety/notes	References
Microbiota Transfer Therapy (MTT): vancomycin + bowel cleanse + FMT	Open-label	18	∼80% reduction in GI symptoms; improved ABC and SRS scores at 8 weeks, persisted at 2 years	Increased *Bifidobacterium*, *Prevotella*, *Desulfovibrio*; donor engraftment	No serious AEs; long-term benefit	(Kang et al., 2017; Kang et al., 2019; Nirmalkar et al., 2022)
Washed microbiota transplantation (WMT)	Prospective	24	Alleviated sleeping disorders and constipation	↓ *Bacteroides*, *Flavonifractor*; ↑ *Prevotella_9*; improved detoxification pathways	Not reported	(Liu et al., 2023)
Colonoscopic FMT vs. control (no FMT)	Open-label controlled	30 (15 FMT)	Improved CARS, Parent Global Impression, GI symptoms; no significant difference in ADOS primary endpoint	Not reported	3 mild self-limited events; no serious AEs	(Varnas et al., 2025)
Probiotics (*Bifidobacterium* + FOS)	Open-label	26	Improved CARS and GI symptoms; partially normalized hyperserotonemia and dopamine metabolism	↑ *Bifidobacterium longum*; ↓ *Clostridium*; ↑ fecal SCFAs	Well tolerated	(Wang et al., 2020)
*B. animalis* subsp. *lactis* Probio-M8 + medium-carbohydrate diet	Open-label	53	Improved CARS and GI symptom scores	↑ *Akkermansia muciniphila*, *Fusicatenibacter*; ↓ *Blautia obeum*; modulated lysine/GABA/vitamin B3 pathways	Pilot study	(Li et al., 2024)
Modified ketogenic diet	Prospective	7	Improved sociability (previously reported); ↓ plasma IL-12p70, IL-1β	↑ butyrate kinase gene expression; altered BDNF-associated miRNAs	Small sample; open-label	(Allan et al., 2024)
*Bifidobacterium infantis* + bovine colostrum	Randomized, double-blind, cross-over	8	Reduced GI symptoms and aberrant behaviors in some participants	Trends in microbiota; decreased IL-13 and TNF-α production	Well tolerated	(Sanctuary et al., 2019)

AE, adverse event; ABC, Aberrant Behavior Checklist; SRS, Social Responsiveness Scale; CARS, Childhood Autism Rating Scale; ADOS, Autism Diagnostic Observation Schedule. Autism Spectrum Disorder, ASD.

Potential risks of FMT include transmission of multidrug-resistant organisms, induction of adverse inflammatory responses, and, in rare cases, procedure-related complications such as perforation or aspiration. None of the trials reviewed reported serious adverse events, but long-term safety data are lacking. Donor variability in microbial composition and the absence of standardized preparation protocols remain major challenges for reproducibility and comparability across studies. Ethical considerations include informed consent for pediatric FMT, management of potential long-term risks, and the need for rigorous oversight. Future perspectives for FMT in ASD include rigorous donor screening, the use of encapsulated formulations to improve tolerability, the identification of responder-associated microbial signatures (e.g., baseline Prevotella abundance) to enable personalized treatment, and the combination of FMT with prebiotics or dietary modifications to enhance engraftment and durability of effects.

### 5.4 Probiotic, prebiotic, and dietary interventions

Beyond FMT, several studies have examined probiotics, prebiotics, and dietary interventions as more accessible and less invasive approaches. Wang et al. (2020) administered a combination of probiotics (primarily *Bifidobacterium*) and FOS to 26 children with ASD for several weeks. After treatment, fecal *Bifidobacterium* longum increased, while *Clostridium* decreased. Autism severity (assessed by CARS) and GI symptoms significantly improved. Baseline metabolic abnormalities—hyperserotonemia (elevated serotonin) and dopamine metabolism disorder (low homovanillic acid)—were partially normalized, and SCFA levels (acetic, propionic, butyric) increased toward control values (Wang et al., 2020). A large-scale synbiotic study by Phan et al. (2024) enrolled 170 children with ASD and 123 neurotypical controls, administering a multi-strain probiotic combined with prebiotic fibers for 6 months (Phan et al., 2024). The treatment significantly improved GI symptoms and reduced aberrant behaviors, accompanied by increased fecal *Bifidobacterium* and *Lactobacillus* and decreased *Clostridium* (Phan et al., 2024). Li et al. (2024) used a specific probiotic strain, *Bifidobacterium animalis* subsp. *lactis Probio-M8*, combined with a medium-carbohydrate diet (approximately 40–45% of total daily calories from carbohydrates, lower than typical Chinese diet) in 53 children with ASD over 3 months. This intervention significantly improved CARS and GI symptom scores, increased fecal abundances of *Akkermansia muciniphila, Fusicatenibacter saccharivorans*, and *Sutterella* sp., and decreased *Blautia obeum*. Metabolomic analysis revealed modulation of pathways related to lysine metabolism, glutamate and GABA metabolism, polyunsaturated fatty acids, and vitamin B3 (Li et al., 2024). In a randomized, double-blind, placebo-controlled pilot study, Hrnciarova et al. (2024) supplemented 20 children with ASD with a Juvenil (a biological response modifier containing inactivated bacterial components, including *Lactobacillus* and *Bifidobacterium* strains, and yeast extracts) for 3 months and found that the intervention converted the gut microbiome toward a more neurotypical profile and positively modulated autism symptoms (Hrnciarova et al., 2024). Sanctuary et al. (2019) conducted a small randomized, double-blind, cross-over trial of *Bifidobacterium infantis* combined with bovine colostrum (as a prebiotic source) in 8 children with ASD and GI comorbidities. The combination treatment was well tolerated, and some participants experienced reductions in GI symptoms and aberrant behaviors, possibly mediated by decreased IL-13 and TNF-α production (Sanctuary et al., 2019).

Prebiotics alone have also been explored. Saxami et al. (2023) used an *in vitro* fermentation system to test the effects of edible mushrooms (*Pleurotus eryngii* and *P. ostreatus*) on fecal microbiota from ASD and neurotypical children. Both mushrooms increased *Bifidobacterium* and butyrate production. Notably, *P. eryngii* also elevated total bacteria, *Bacteroides*, and *Faecalibacterium prausnitzii* in the ASD group, suggesting that mushroom-derived prebiotics may selectively promote beneficial taxa (Saxami et al., 2023). Dietary interventions beyond prebiotics include the modified KD. Allan et al. (2024) followed 7 children with ASD on a KD for 4 months and observed decreased plasma levels of pro-inflammatory cytokines (IL-12p70, IL-1β) and brain-derived neurotrophic factor (BDNF), increased expression of the butyrate kinase gene in stool, and altered BDNF-associated miRNAs. These changes were accompanied by previously reported improvements in sociability, suggesting that the KD may reduce inflammation and reverse gut dysbiosis (Allan et al., 2024). A protocol for a large randomized controlled trial of a probiotic mixture [Vivomixx^®^; it should be noted that post-2016 the VSL#3 probiotic formulation differs from the De Simone Formulation, which was commercially available under the trademark VSL#3^®^ only until 2016 (De Simone, 2018)] (De Simone, 2018) in 100 preschoolers with ASD has also been published, with plans to assess clinical, biochemical, and neurophysiological outcomes (Santocchi et al., 2016). In addition to probiotics and prebiotics, certain phytocompounds (e.g., curcumin, resveratrol) have shown preclinical effects on gut microbiota and neuroinflammation in animal models of ASD, but clinical evidence in children remains preliminary and is not yet sufficient for recommendation.

### 5.5 Summary of translational evidence and future trial requirements

In summary, a convergence of evidence from animal models and early-phase clinical trials indicates that gut microbiota dysbiosis plays a causal role in ASD pathophysiology and that restoring a healthy microbiota via FMT, probiotics, prebiotics, or dietary modifications can ameliorate both GI and behavioral symptoms. However, most human studies remain open-label or underpowered, and the only controlled trial of FMT to date did not meet its primary ADOS endpoint. Therefore, while the therapeutic potential is substantial, large-scale, double-blind, placebo-controlled trials with standardized outcome measures and long-term follow-up are urgently needed to establish definitive efficacy and safety profiles before these interventions can be recommended for routine clinical use.

## 6 Diagnostic and subtyping potential of gut microbial biomarkers

A key translational question is whether gut microbial features can serve as non-invasive biomarkers for ASD diagnosis or for stratifying clinical subtypes. Several studies have explored this possibility with promising but preliminary results. For instance, bacterial genomic structural variations (SVs)—rather than mere relative abundances—have shown high discriminatory power. Liu et al. (2026) identified 100 SVs associated with ASD, and a diagnostic model combining nine SVs and three bacterial species achieved an area under the curve (AUC) of 81.1%, outperforming models based solely on species abundance (72.3%) (Liu et al., 2026). Similarly, Chen et al. (2026) integrated metagenomic and metabolomic data from over 1,100 children, uncovering 1,369 single-nucleotide variants, 233 indels, and 195 structural variants across 35 species that distinguished ASD from controls (Chen et al., 2026). These findings suggest that microbial genomic variation, an underappreciated dimension of dysbiosis, could provide stable, individualized biomarkers.

Beyond genomics, host epigenetic signatures in fecal DNA offer another layer of diagnostic information. Cuomo et al. (2023) demonstrated that whole-genome methylation analysis of human fecal DNA could discriminate ASD children from healthy controls, with differentially methylated regions enriched in inflammation-related genes. Notably, both epigenetic and microbial ages were accelerated in ASD, implying premature aging of the gut ecosystem (Cuomo et al., 2023). Such multi-omics approaches–combining metagenomics, metabolomics, and host epigenetics–are likely to yield more accurate and mechanistically informative biomarkers than any single data type.

However, several hurdles remain before clinical implementation. First, most biomarker studies are cross-sectional and lack validation in independent, large cohorts. Second, the substantial heterogeneity across populations (e.g., Chinese *Escherichia-Shigella* increase versus European Bacteroides increase) raises questions about generalizability. Third, urinary metabolites such as p-cresol and HPHPA, initially proposed as markers of *Clostridium* overgrowth, have shown limited specificity (Daneberga et al., 2022). Therefore, while microbial and epigenetic signatures hold promise, they should currently be viewed as research tools rather than ready-for-clinic diagnostics. Future work must prioritize multi-center, longitudinal validation studies with standardized protocols (Table 5).

**TABLE 5:** Multi-omics and biomarker studies for ASD diagnosis and subtyping.

Approach	Sample type	Key discriminative features	Diagnostic/ classification performance	Biological insight	References
Metagenomic structural variants (SVs)	Stool (fecal metagenomics)	100 SVs associated with ASD; model combining 9 SVs + 3 bacterial species	AUC = 81.1% (vs. 72.3% for species abundance alone)	SVs outperform relative abundance as stable microbial biomarkers	(Liu et al., 2026)
Integrated metagenomics and metabolomics	Stool + plasma	35 species, 213 genes, 28 pathways, 99 metabolites; 1,369 SNVs, 233 indels, 195 SVs	Discriminated ASD from controls in > 1,100 children	Mediation analysis: metabolites link microbial variants to ASD phenotype	(Chen et al., 2026)
Host fecal DNA methylation	Stool (human DNA)	Differentially methylated regions in inflammation-related genes; accelerated epigenetic and microbial age	Discriminated ASD children from healthy controls	Human fecal DNA derived mainly from immune cells; age acceleration in gut ecosystem	(Cuomo et al., 2023)
Integrative meta-omics (metagenomics, metabolomics, cytokines)	Stool + plasma	“TNFα-sphingolipids-steroid hormones” axis	Not reported (mechanistic proposal)	TNFα correlates with microbial sphingolipid metabolism, negatively correlates with steroid hormones	(Shao et al., 2024)
Urinary organic acids	Urine	p-Cresol, HPHPA	Not reliable as specific markers for *Clostridium* overgrowth	Elevated p-cresol may reflect increased Firmicutes, not solely *Clostridium*	(Daneberga et al., 2022)

AUC, area under the curve; SNV, single-nucleotide variant; HPHPA, 3-(3-hydroxyphenyl)-3-hydroxypropionic acid; ASD, Autism Spectrum Disorder.

## 7 Comparison of gut dysbiosis between ASD and other neurodevelopmental disorders

Understanding whether the gut dysbiosis observed in ASD is disorder-specific or shared across neurodevelopmental conditions is crucial for both mechanistic insight and therapeutic targeting. The following comparison focuses on ADHD and tic disorders as neurodevelopmental conditions. Anorexia nervosa (AN), although primarily classified as a psychiatric disorder rather than a neurodevelopmental condition, is included here because individuals with ASD have an elevated risk of developing AN and because the growing literature on gut dysbiosis in AN offers interesting comparative insights. Comparative studies including ADHD, AN, and tic disorders reveal both commonalities and differences.

Soltysova et al. (2025) directly compared gut microbiota in children with ASD, ADHD, and AN (included as a psychiatric comparator). Common patterns across all three disorders included a higher *Bacteroidetes/Firmicutes* ratio, reduced *Bifidobacterium*, and decreased *Faecalibacterium* (in ADHD and AN). However, ASD uniquely exhibited elevated *Bacteroidetes* and diminished *Actinobacteriota* and *Ruminococcu*s; ADHD showed reduced *Firmicutes*; AN displayed decreased *Firmicutes* and increased *Proteobacteria*, *Cyanobacteria*, and *Verrucomicrobiota* (Soltysova et al., 2025). These findings suggest that while some dysbiotic features (e.g., loss of *Bifidobacterium*) may reflect general neurodevelopmental vulnerability, other taxa are disorder-specific and could potentially serve as differential diagnostic markers.

In ADHD specifically, Wang et al. (2022) found no significant differences in α- or β-diversity but identified three differentially abundant genera (*Agathobacter, Anaerostipes, Lachnospiraceae*) and lower plasma TNF-α levels compared to controls (Wang et al., 2022). A meta-analysis by Fakruddin et al. (2026) further emphasized that ASD is associated with high *Clostridium* and low *Bifidobacterium*, whereas ADHD is associated with low *E. coli, high Clostridia, Firmicutes, Bifidobacterium, Akkermansia*, and several pathogens (Fakruddin et al., 2026). For tic disorders, Wu et al. (2026) used Mendelian randomization and 16S sequencing to identify protective (*Anaerotruncus, Butyrivibrio, Ruminococcaceae* UCG-002) and risk-associated (*Dialister, Ruminiclostridium* 6) genera (Wu et al., 2026). These results support the concept that distinct gut microbial signatures may accompany different neurodevelopmental conditions (ADHD and tic disorders), while AN shows some overlapping features.

From a clinical perspective, these overlaps and distinctions have two important implications. First, interventions that broadly restore beneficial bacteria (e.g., *Bifidobacterium, Faecalibacterium*) might be beneficial across multiple disorders, but the optimal microbial targets may differ. Second, when using gut microbiota as a diagnostic aid, it will be necessary to differentiate ASD from ADHD or AN, which may require disorder-specific panels. Currently, the evidence base is too limited to draw firm conclusions, and larger head-to-head comparative studies are urgently needed.

## 8 Discussion

The present systematic review collectively demonstrating that gut microbiota dysbiosis is consistently associated with ASD across diverse populations and age groups. The most robust microbial signatures include decreased *Bifidobacterium* and increased *Clostridium*, *Bacteroides*, and *Faecalibacterium*, although geographic, age-, and sex-specific variations exist. Beyond taxonomic shifts, the gut virome and mycobiome are also altered, with enrichment of *Candida albicans* and *Clostridium* phages. Metabolomic and mechanistic studies reveal disrupted tryptophan–serotonin metabolism, reduced SCFAs (particularly butyrate), and elevated neuroinflammatory cytokines (TNF-α, IL-6, CRP). Animal models using FMT have established causality, and early-phase clinical trials indicate that modulating the gut microbiota via FMT, probiotics, prebiotics, or dietary interventions can improve both GI and behavioral symptoms. In this Discussion, we integrate these findings into a broader context, critically examine the potential of microbial biomarkers for ASD diagnosis and subtyping, compare ASD-associated dysbiosis with that of other neurodevelopmental disorders, address the major limitations of current research, and propose concrete future directions.

### 8.1 Integrated mechanistic pathways and therapeutic strategies

The mechanistic pathways linking gut dysbiosis to ASD pathophysiology are now well-supported by metabolomic, immunological, and animal model data. Children with ASD exhibit significantly reduced fecal SCFAs (especially butyrate), which correlates with depletion of butyrate-producing bacteria such as *Faecalibacterium prausnitzii* and *Eubacterium rectale* (Bojović et al., 2020). The tryptophan- serotonin pathway is also disrupted, with hyperserotonemia and altered expression of TPH1, TPH2, and SERT in both human and animal studies (Wang et al., 2020; Xiao et al., 2021). Concurrently, elevated pro-inflammatory cytokines (TNF-α, IL-6, CRP) and fecal calprotectin indicate systemic and intestinal inflammation (Iovene et al., 2017; Lin et al., 2024). These three interconnected mechanisms—SCFA deficiency, serotonin dysregulation, and neuroinflammation—are proposed to converge on gut barrier dysfunction and vagal signaling alterations, ultimately affecting brain development and behavior (Figure 1).

### 8.2 Therapeutic strategies: evidence and gaps

Regarding therapeutic strategies, FMT has produced the most durable benefits, with open-label trials showing sustained improvements in GI and behavioral symptoms for up to 2 years (Kang et al., 2019). Probiotic and prebiotic interventions, particularly those combining *Bifidobacterium* strains with FOS or medium- carbohydrate diet, have demonstrated significant but more modest effects (Li et al., 2024; Wang et al., 2020). However, the only controlled FMT trial to date failed to meet its primary ADOS endpoint, and most studies lack blinding and placebo control (Varnas et al., 2025). Therefore, while the mechanistic rationale is strong, the clinical evidence for routine use remains preliminary. Future trials should stratify patients by baseline microbiota profiles and inflammatory markers to identify responders.

### 8.3 Major limitations and sources of heterogeneity

Despite the growing body of evidence, several critical limitations must be acknowledged. As highlighted by systematic reviews (Fakruddin et al., 2026; Jurek et al., 2021), the field suffers from substantial methodological heterogeneity, including variations in study design, age ranges, diagnostic criteria for ASD, GI symptom assessment, sample type (stool vs. mucosal biopsies), DNA extraction methods, sequencing platforms (16S rRNA vs. shotgun metagenomics), and bioinformatic pipelines. Such heterogeneity hampers direct comparisons and meta-analyses, and may explain why some taxa show inconsistent directions across individual studies.

Confounding factors are another major concern. Diet, antibiotic use, mode of delivery, breastfeeding, and geographic location all influence gut microbiota composition. Although some studies attempted to control for these variables, many did not. For instance, Wan et al. (2022) reported that diet showed no correlation with microbiome in their Chinese cohort, but this may not apply to Western populations where dietary patterns differ more markedly (Wan et al., 2022). Moreover, most studies are cross-sectional, making it impossible to determine whether dysbiosis precedes ASD or results from ASD-related behavioral differences (e.g., selective eating) or medication use. Longitudinal studies starting from infancy are rare but essential.

Small sample sizes remain pervasive. Many included fewer than 50 participants per group, leading to underpowered and potentially spurious findings. Large cohort studies (*n* > 100 per group) are still limited (Wan et al., 2022; Xie et al., 2022). Sex-specific analyses are rarely performed despite known sex differences in ASD prevalence and clinical presentation; when performed, they reveal meaningful differences (De Sales-Millán et al., 2024; Reeves et al., 2024; Tamana et al., 2021). Similarly, age-stratified analyses are underreported, yet the work by Kadiyska et al. (2025) demonstrates that the youngest children (0–4 years) exhibit the greatest microbiota deviations (Kadiyska et al., 2025).

Finally, intervention trials—particularly those involving FMT or probiotics—are predominantly open-label or lack adequate blinding and placebo control. The only published controlled trial of FMT for ASD did not meet its primary ADOS endpoint, although secondary outcomes improved (Varnas et al., 2025). Publication bias may also exist; a meta-analysis noted that the *Bifidobacterium* difference might be influenced by reporting bias (Andreo-Martínez et al., 2022). These limitations underscore the need for more rigorous, pre-registered, and well-powered studies.

### 8.4 Future directions: toward mechanistic understanding and precision therapeutics

Based on the above findings and limitations, we propose several priority areas for future research. First, longitudinal cohort studies starting from pregnancy or birth, with repeated sampling of microbiota, metabolome, and dietary records, are essential to establish temporal causality. Such studies should also collect information on antibiotics, infections, and stress—all of which can shape the gut–brain axis. The EPIFLORE (Rozé et al., 2020) and Tian et al. (2026) studies provide promising models in preterm infants, but similar efforts are needed in term children. Second, stratification by age, sex, GI phenotype, and ASD severity should become standard. The finding that mild ASD microbiota more closely resembles that of neurotypical children than severe ASD (Zeng et al., 2025) suggests that microbial signatures could correlate with symptom burden. Sex-stratified analyses are particularly needed given the male predominance in ASD and the sex-specific microbial associations reported (De Sales-Millán et al., 2024; Reeves et al., 2024). Third, double-blind, placebo-controlled randomized trials of FMT, probiotics, and dietary interventions are urgently required. Future trials should use standardized, validated outcome measures (including ADOS, CARS, and GI rating scales) and include long-term follow-up ( ≥ 1 year). The lack of effect on ADOS in the Varnas et al. trial (Varnas et al., 2025) highlights the need for careful selection of primary endpoints and possibly for combining microbiome modulation with behavioral therapies. Fourth, multi-omics integration—metagenomics, metabolomics, host genetics, epigenetics, and immune profiling—should be applied in large cohorts to identify causal pathways and therapeutic targets. The “TNFα-sphingolipids-steroid hormones” axis proposed by Shao et al. (2024) and the vitamin B6 metabolism link identified by Zheng et al. (2024) are examples of mechanistically plausible, testable hypotheses arising from integrative analyses. Fifth, exploration of the gut virome, mycobiome, and bacterial SVs should be expanded. These components are largely neglected but may hold key roles in maintaining dysbiosis and influencing host metabolism. For instance, phage therapy could potentially be used to selectively reduce pathobionts, and antifungal strategies might benefit a subset of ASD patients with Candida overgrowth (Iovene et al., 2017; Retuerto et al., 2024). Lastly, harmonization of protocols across research groups—including standardized sampling, sequencing, and analysis pipelines—would greatly enhance comparability and enable large-scale meta-analyses. Initiatives such as the International Microbiome Consortium for ASD could facilitate data sharing and replication.

## 9 Conclusion

This systematic review demonstrates that gut microbiota dysbiosis is consistently associated with ASD, characterized by reduced *Bifidobacterium* and *Akkermansia* and increased *Clostridium*, *Bacteroides*, *Escherichia-Shigella*, and *Candida albicans*, with significant age, sex, geographic, and clinical subgroup variations. Mechanistically, dysbiosis leads to reduced SCFAs (especially butyrate), disrupted tryptophan-serotonin metabolism (hyperserotonemia), and elevated neuroinflammatory cytokines (TNF-α, IL-6, CRP), which collectively impair gut barrier function and signal via the gut–brain axis to influence behavior. Causal evidence from animal FMT models confirms that ASD microbiota can directly induce autistic-like behaviors. Therapeutically, FMT, probiotics, prebiotics, and dietary interventions have shown encouraging results in early-phase trials, improving both GI and behavioral symptoms; however, the only controlled FMT trial failed to meet its primary ADOS endpoint, and most studies lack blinding and placebo control. Emerging biomarkers—bacterial structural variants and host fecal DNA methylation—demonstrate diagnostic promise (AUC up to 81.1%) but require validation. In conclusion, gut dysbiosis plays a causal role in ASD, and microbiome-based interventions represent a rational therapeutic avenue, but large-scale, double-blind, placebo-controlled trials are urgently needed before clinical translation. Beyond microbiome-focused strategies, emerging theranostic approaches—including AI-assisted screening, neuroimaging-guided interventions, precision medicine, and nanotheranostics—hold promise for future ASD management, although their integration with gut microbiota research remains to be explored.
